# Supplementary material for: Content-rich biological network constructed by mining PubMed abstracts
Source: BMC Bioinformatics. 2004 Oct 8;5:147. doi: 10.1186/1471-2105-5-147 (PMC528731; doi:10.1186/1471-2105-5-147)
Supplement: Additional File 2 — The original results of the above study (non-essential files are deleted to keep the file size under the limit set by BMC bioinformatics). [file 1471-2105-5-147-S2.bz2 › chilibotAdditionalFile2/dip20/158ID7685215E560/html/FOS_TBP.html]

 


 **FOS** and **TBP** 
  
Found 30 abstracts in PubMed, retrieved 20.  
 

 What does Google say? 
 PDF only 
| .edu only 

---

**Interactive relationship** (e.g. stimulation, inhibition, etc)

**Neutral relationship**- The topical application of 50 microg cm2 BaP  [ **TBP** ]  to EpiDerm resulted in the accumulation of BaP  [ **TBP** ]  DNA adducts and c  **fos**  and p53 proteins as evidenced by immunohistochemical localization.  Ref: 9920731 Biochem Biophys Res Commun, 1999
- To examine protein protein interactions with cellular proteins at the visna virus promoter, we used an in vitro protein affinity chromatography assay and electrophoretic mobility shift assay, in addition to an in vivo two hybrid assay, to show that the visna virus Tat protein specifically interacts with the cellular transcription factors  **Fos**  and Jun and the basal transcription factor  **TBP**  TATA binding protein  [ **TBP** ] .  Ref: 9847304 J Virol, 1999

**Non-interactive relationship** (e.g. studied together, co-existance, homology, etc.)

- Strikingly, the first modification observed is methylation of histone H3 lysine 4, which correlates with the binding of the SET9 methyltransferase and the assembly of a complex consisting of c Jun, c  **Fos** , TATA binding protein  [ **TBP** ] , and RNA polymerase II.  Ref: 12588998 Mol Cell Biol, 2003
- BaP  [ **TBP** ]  MeoA cells acquired the greatest proliferative enhancement and exhibited unregulated c jun and c  **fos**  gene expression under growth arrested and serum stimulated conditions.  Ref: 7487084 Arch Biochem Biophys, 1995
- We find that the ability to stabilize the  **TBP**  TFIIA complex on the hsp70 and c  **fos**  TATA elements, and not on the SV40 early and E2A TATA elements, correlates with the ability or inability to activate promoters containing these TATA elements.  Ref: 9632777 Mol Cell Biol, 1998
- However, the levels of p53 and ras proteins and the levels of p53, c myc and c  **fos**  transcripts in the HOK 16B BaP  [ **TBP** ]  T cells were not different from those in the HOK 16B BaP  [ **TBP** ]  cells.  Ref: 7784058 Oncogene, 1995
- Mutation of either Tyr248, Tyr357, or Tyr462 to phenylalanine reduced transcription from a c  **fos**  promoter relative to wild type BAP  [ **TBP** ]  TFII I in transfected COS 7 cells, consistent with the interpretation that phosphorylation at these sites contributes to transcriptional activation.  Ref: 11373296 J Biol Chem, 2001
- These includes nuclear factor,  **FOS**  related antigen 1, cytoplasmic dynein light chain, replication factor C, nucleoside diphosphate kinase B, UV excision repair protein, insulin like growth factor receptor II, transcription initiation factor TFIID  [ **TBP** ]  subunit, growth factor receptor bound protein 2, UV excision repair protein, glutathione peroxidase, Y box binding protein 1 and heat shock protein 86.  Ref: 10946852 Life Sci, 2000
- c  **fos**  and c Ha ras gene expression was enhanced in liver, but not kidney, slices by BaP  [ **TBP** ] . c jun mRNA levels were decreased in liver and kidney slices, although the effect was earlier 0.5 h in liver slices compared to kidney slices.  Ref: 9852999 Toxicol Appl Pharmacol, 1998
- The association between Tat,  **Fos** , and Jun would position Tat proximal to the viral TATA box, where the visna virus Tat activation domain could contact  **TBP**  to activate viral transcription.  Ref: 9847304 J Virol, 1999
- It is the regulatory subunit of the DNA dependent protein kinase that phosphorylates many proteins, including SV 40 large T antigen, p53, RNA polymerase II, RP A, topoisomerases, hsp90, and many transcription factors such as c Jun, c  **Fos** , oct 1, sp 1, c Myc, TFIID  [ **TBP** ] , and many more.  Ref: 10755664 Crit Rev Biochem Mol Biol, 2000
